# Outlasting a Rare Duodenal Angiosarcoma

**DOI:** 10.7759/cureus.5097

**Published:** 2019-07-08

**Authors:** Sindhura Kolli, Owen Chan, C. Galen Choy, Mel A Ona

**Affiliations:** 1 Clinical Obesity Medicine, NYU Langone Health, New York, USA; 2 Pathology, Pali Momi Medical Center, Affiliate of Hawaiʻi Pacific Health, Honolulu, USA; 3 Oncology, Pali Momi Medical Center, Affiliate of Hawaiʻi Pacific Health, Honolulu, USA; 4 Gastroenterology, Pali Momi Medical Center, Affiliate of Hawaiʻi Pacific Health, Honolulu, USA

**Keywords:** duodenal angiosarcoma, angiosarcoma, gastroenterology, small intestine angiosarcoma

## Abstract

With current life expectancies exceeding 78 years on average, to be confronted with the discovery of a rare cancer often found in advanced stages is a startling devastation. Angiosarcoma of the intestine is a rare and aggressive tumor that is not often considered in the differential diagnosis of intestinal obstruction. Once found and accurately diagnosed, it is a bewildering race against time as its median survival time is 150 days from diagnosis. This case report details a rare small intestinal angiosarcoma with its host surpassing current epidemiological standards of survival time despite only being eligible for chemotherapy.

## Introduction

Due to its rarity, non-specific presenting symptoms, difficulty in diagnosing, aggressive sequelae, and high mortality rate, intestinal angiosarcomas define a perfect storm of a medical disaster. This case report details a case of a stage IV angiosarcoma that occurred in a rare and unusual location of the small intestine with an unexpected outcome of the patient living four times the average lifespan from diagnosis despite being treated with chemotherapy alone.

## Case presentation

**Figure 1:**
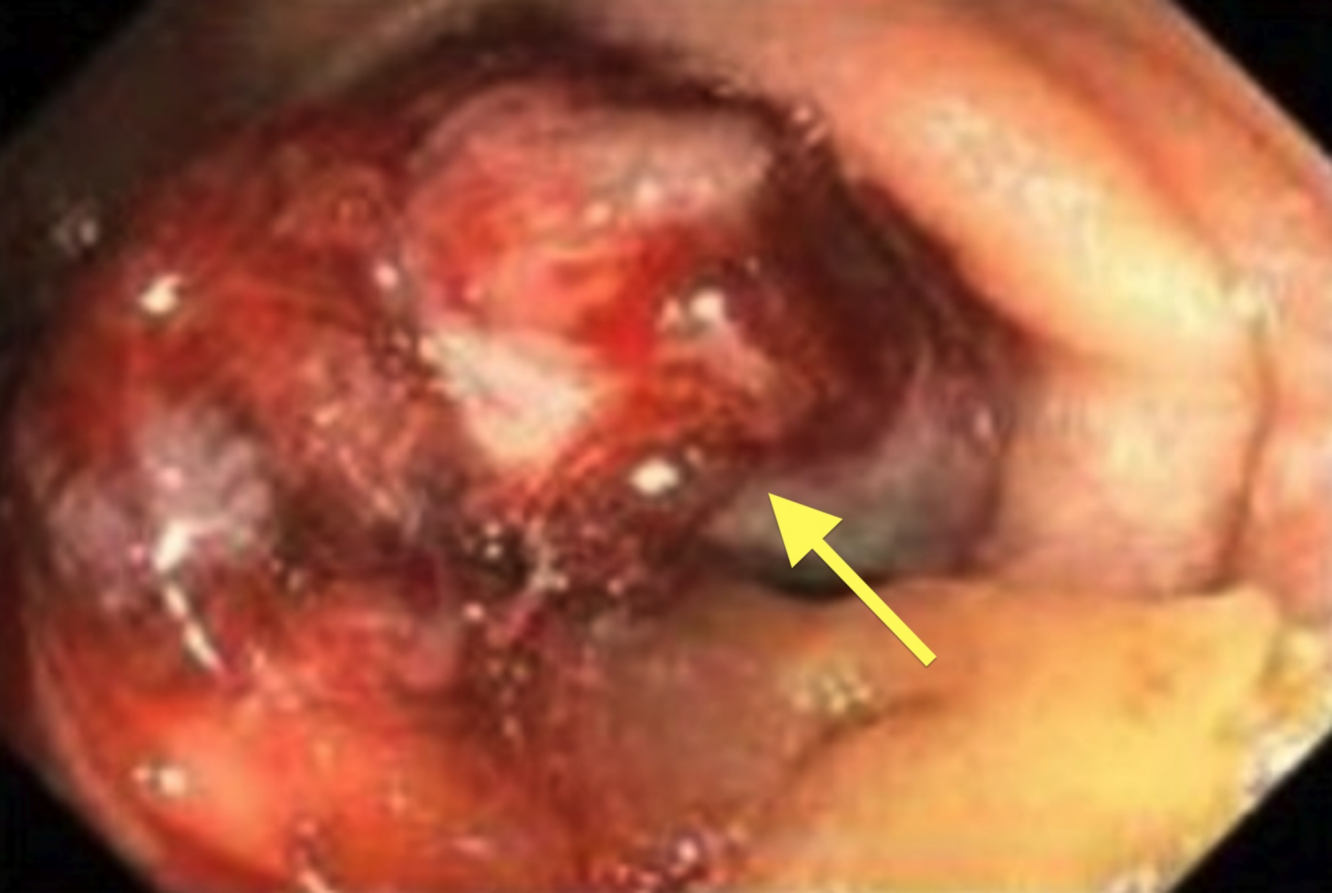
Infiltrative fungating, circumferential, 2 cm angiosarcoma in the third part of the duodenum causing partial obstruction

A 76-year-old male patient presented with complaints of epigastric abdominal pain with nausea, fatigue, and unintentional weight loss of 12 pounds over one week with normal bowel movements. His family history is significant for a sister with gastric cancer. An initial computed tomography (CT) scan without contrast displayed a circumferential, mass-like thickening at the distal transverse duodenum with abdominal mesenteric and retroperitoneal lymphadenopathy. A subsequent esophagogastroduodenoscopy demonstrated an infiltrative, fungating, circumferential, 2 cm mass in the third part of the duodenum that caused a partial obstruction where the scope could not traverse (Figure 1).

**Figure 2:**
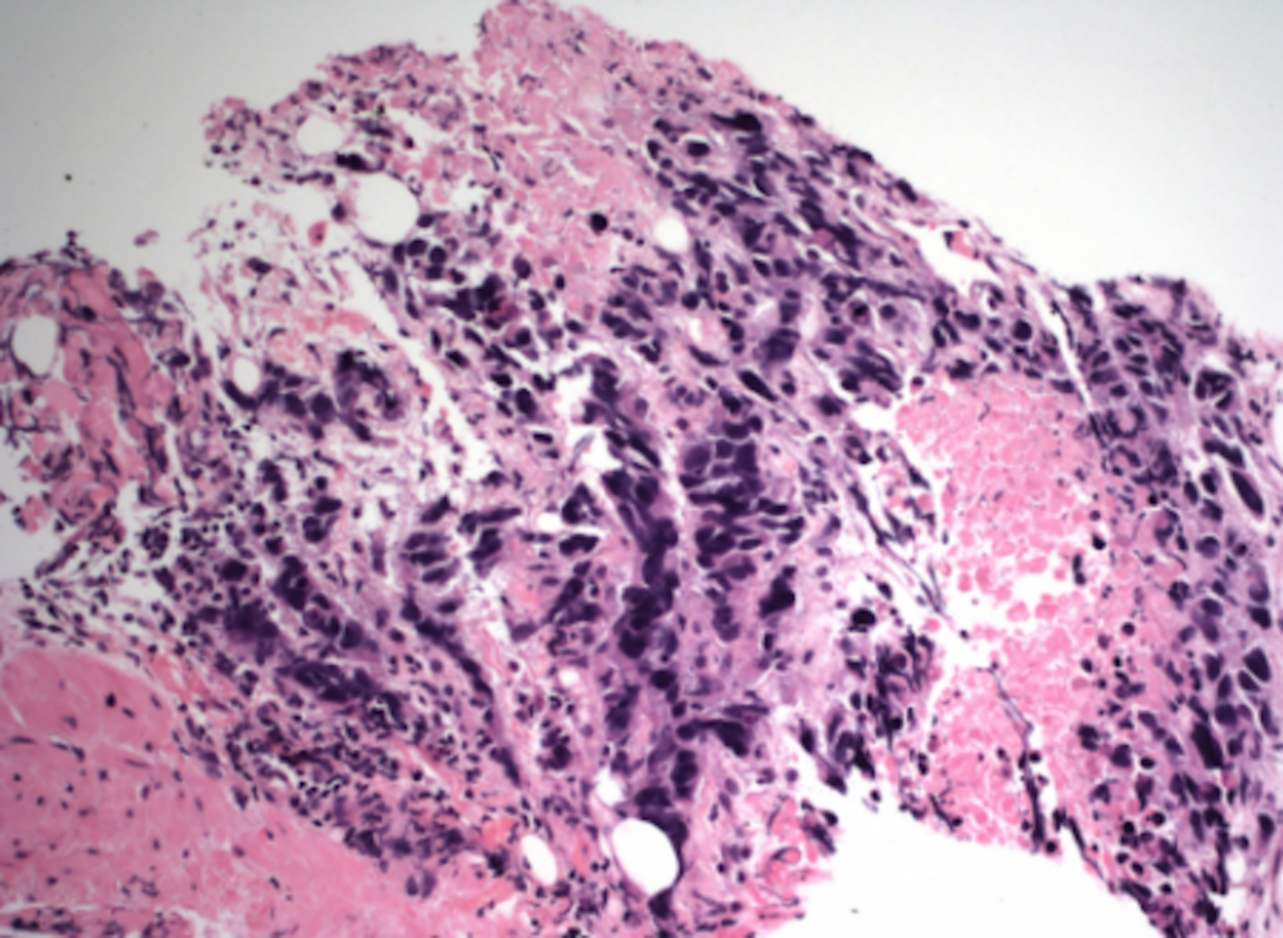
H&E stain (magnification 200x) revealing duodenal angiosarcoma H&E, hematoxylin and eosin

Biopsies of the mass, as well as the left peri-aortic lymph node, revealed poorly differentiated, malignant cells infiltrating the small intestine mucosa via hematoxylin and eosin staining (Figure 2).

**Figure 3:**
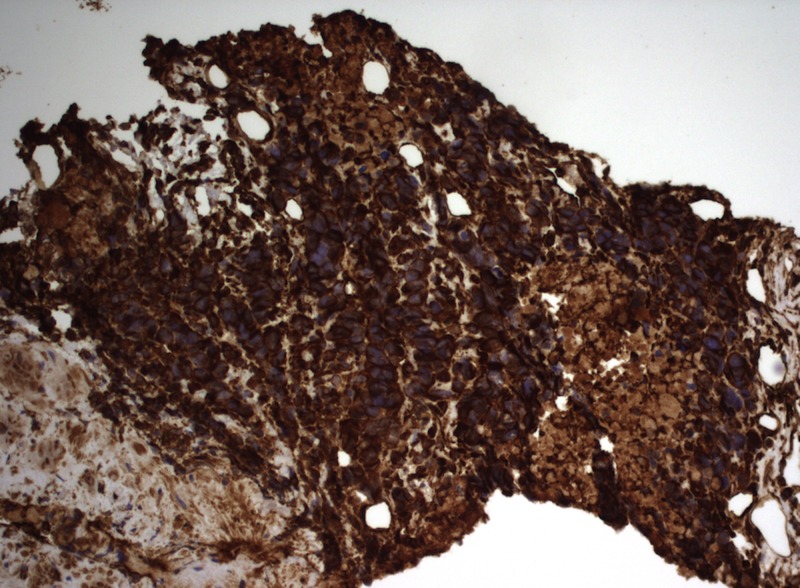
CD31 staining positive for angiosarcoma

To determine the tissue origin of the cells, immunohistochemistry (IHC) was performed. The malignant cells were positive for vimentin, CD31 (Figure 3), and ETS-related gene (ERG).

**Figure 4:**
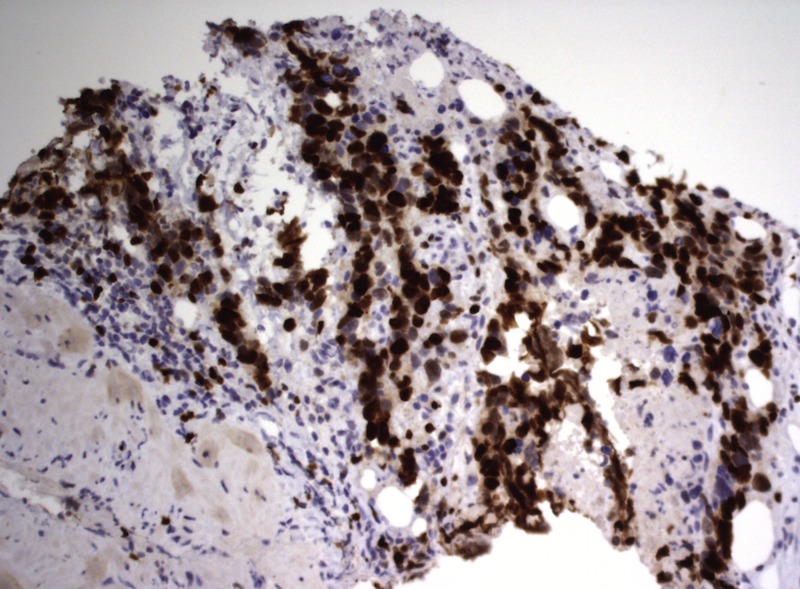
Ki-67 staining indicating cellular proliferation

The cells had equivocal staining for factor 8 and CD34. Collectively, the data indicated a high-grade sarcoma with vascular differentiation. It also demonstrated increased staining for Ki-67, a marker of cellular proliferation (Figure 4).

**Figure 5:**
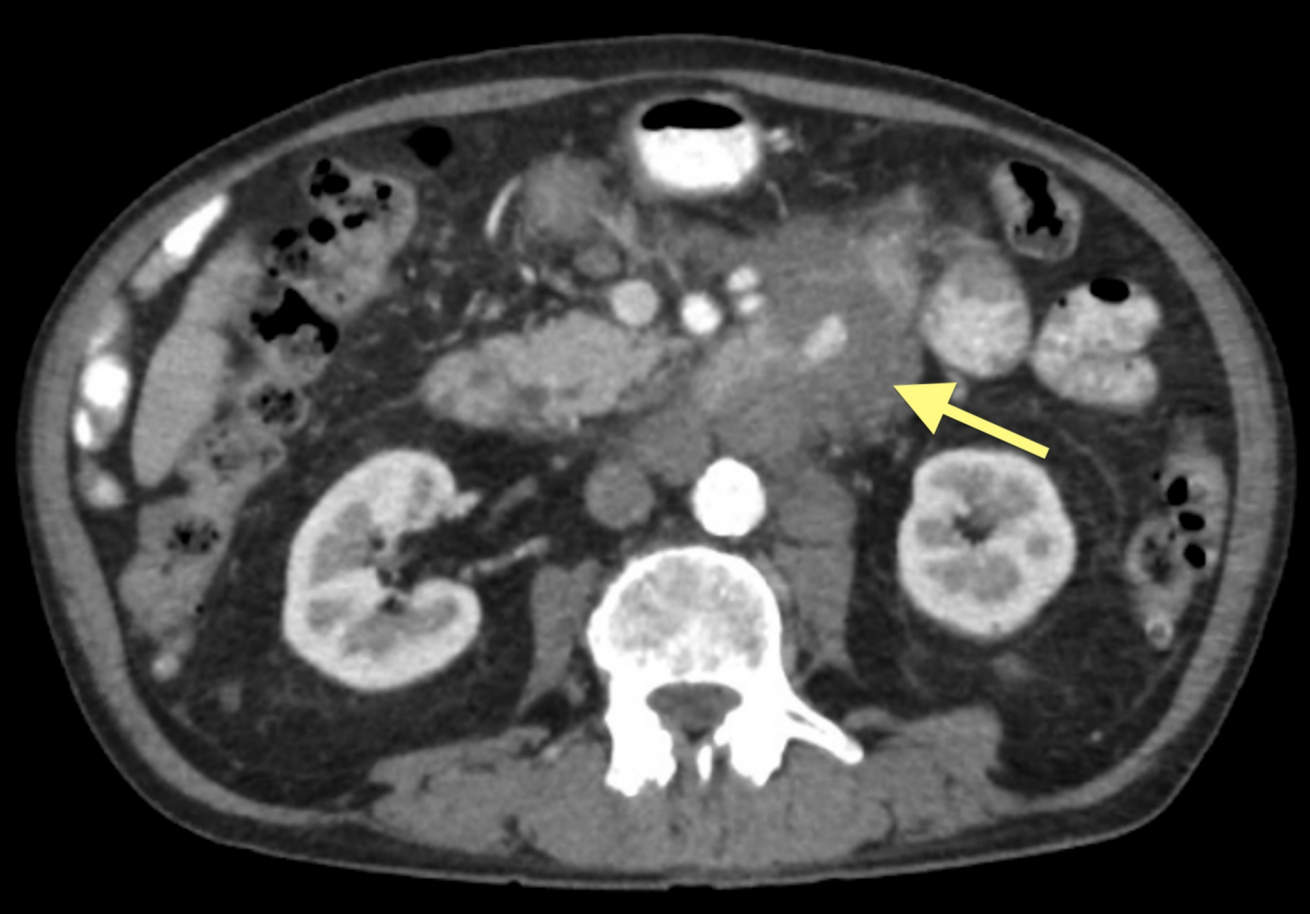
CT of abdomen and pelvis with contrast demonstrating duodenal angiosarcoma CT, computed tomography

The patient underwent seven rounds of paclitaxel chemotherapy from January to August 2018. A CT performed in March 2018 showed a decrease in the duodenal mass size, stable pulmonary nodules, and decreases in the sizes of the hepatic masses and lymphadenopathy. However, a repeat CT in August 2018 showed an increase in the sizes of the duodenal mass, pulmonary metastases, and the diffuse lymphadenopathy (Figure 5).

It also exposed a partial filling defect in the superior mesenteric vein, which was presumed to be vessel tumor invasion. A transthoracic echocardiogram disclosed a markedly decreased left ventricular ejection fraction of 20% with severe hypokinesis of the apex, anterior wall, and septum. He was hospitalized with acute respiratory failure, non-ST elevation myocardial infarction, congestive heart failure exacerbation, and pneumonia while undergoing chemotherapy. Upon these findings, both the patient and oncologist agreed to no longer proceed with chemotherapy, and palliative care was offered with monthly follow-up.

## Discussion

Intestinal angiosarcomas are malignant sarcomas derived from endothelial cells, and they make up 1%-2% of all sarcomas [1-2]. Known for their aggressive sequelae and high mortality rate, their rarity confounds epidemiology, making diagnosis arduous. Angiosarcomas have a proclivity for skin and subcutaneous tissue, but occasionally they can occur in visceral organs and bones. They seldom appear in the small intestine primarily or metastatically. Within the small intestine, angiosarcomas are found more commonly in the ileum and jejunum, with only 4.3% of the previously recorded cases in the literature being found in the duodenum only [1]. This makes our case singularly unique in its unfortunate presentation.

Angiosarcomas are associated predominantly with radiation exposure (with a latency period of ~9.5 years from exposure), environmental toxins, and genetics [3]. It affects men more often than women at a ratio of 1.6:1. Affecting patients at the age of 68.5 years on average, it is usually found in the advanced stages of II or III. This makes resection a difficult possibility, resulting in a poor prognosis and a median survival period of approximately 150 days. Women fare marginally better with an average survival time of 300 days from diagnosis, while median survival time in men is 120 days [1]. Clinical diagnosis remains challenging, as presenting complaints of nausea, vomiting, abdominal pain, gastrointestinal bleeding causing anemia, fatigue and weakness, anorexia, and occasionally abdominal obstruction and perforation are non-specific symptoms, depleting time and increasing healthcare expenditure, as other more likely differentials are pursued. Initial workup includes a CT, MRI, or positron-emission tomography to qualify its presence. Ultrasound, barium study, visceral angiogram, and technetium-99mm red blood cell scans can outline the extent of obstruction and effects on surrounding structures [3]. Definitive diagnosis must be made via endoscopy and biopsy or surgical resection, where the tumor grossly resembles a group of multiple, dark red, grape-like clusters. IHC usually reveals an irregular, anastomosing vasculature lined by atypical endothelial cells. Histology can present in the following three ways: 1) spindle-shaped endothelial cells, 2) epithelioid with large, round or polygonal cells, or 3) pleomorphic cells [3-4]. Even at this stage of diagnosis, the tissue can easily be mistaken for a poorly differentiated carcinoma, lymphoma, or malignant melanoma; therefore, tissue diagnosis is often used in conjunction with IHC. The most commonly relied upon immunostains include von Willebrand factor (vWF), CD31, CD34, vimentin, endothelin-1, vascular endothelial growth factor receptor, friend leukemia integration-1, ERG, and ulex europaeus agglutinin-1 (UEA-1). High diagnostic value markers include vWF, CD31, and UEA-1, while vimentin is associated with accelerated tumor growth and poor prognosis, and elevated Ki-67 is associated with cellular proliferation. However, these markers can be lost with progressive tumor de-differentiation, making melanoma and carcinoma possible differential diagnoses again [1-2,4-5]. Diagnosis is key for treatment and prognosis; but currently available diagnostic modalities are limited, resulting in a murky and arduous process.

Once appropriately diagnosed and staged, many angiosarcomas are treated with complete surgical resection with wide margins for localized tumors or as a palliative measure when metastatic. In cases where surgery was combined with chemotherapy, patients have a marginally longer life span of 420 days versus 96.5 days of extended survival with just surgery alone [1]. Current chemotherapy agents are paclitaxel, docetaxel, vinorelbine, sorafenib, sunitinib, and bevacizumab as per the NCCN guidelines [1-6]. Radiation is never used alone but used in conjunction with chemotherapy and surgery. Due to the drastically reduced life span, recurrence rates are currently unknown [1].

## Conclusions

A stage IV angiosarcoma partially obstructing the lumen of the third part of the duodenum defied norms by being managed by chemotherapy alone, when the current standard involves surgery. This case delved into an extensive review of angiosarcomas, while shedding light on an alternative treatment plan.
